# Intercalary Allograft Reconstruction of the Femur: A Cadaveric Comparison of Intramedullary and Plate Fixation Techniques

**DOI:** 10.1002/jor.70260

**Published:** 2026-08-02

**Authors:** Yazan Kadkoy, Gregory James Schneider, Joseph Anthony Ippolito, Thomas Philip Helbig, Vijay Subramanian, Jonathan Rene Lopez, David N. Paglia, Joseph Benevenia

**Affiliations:** ^1^ Department of Orthopaedics Rutgers‐New Jersey Medical School Newark New Jersey USA

## Abstract

Intercalary reconstruction following diaphyseal tumor resection is advantageous in that they preserve the joint above and below. Recently, intramedullary devices such as intramedullary nails (IMN), photodynamic bone stabilizing system (PBSS), and intercalary endoprosthetic reconstruction (EPR) have become increasingly used. Understanding the tradeoffs associated with each construct as well as characterizing their mechanical stability is essential for making a patient‐specific decision for reconstruction. The mechanical properties of four different constructs used in the reconstruction of segmental defects in a femur model were compared: Double plate (DP) allograft secured by 90–90 plating, allograft secured by IMN and plate fixation, allograft secured by PBSS and plate fixation, as well as an intercalary prosthesis (EPR). Samples were tested in axial, bending and torsional loading, and mechanical properties were compared. The EPR during anterior–posterior bending had 51.63% of the displacement compared to the DP (*p* = 0.0007), and 55% of the creep over 100 cycles (*p* = 0.0126) with a rigidity of 201.3% (*p* = 0.0067). This difference also exists for cyclic torsion where the EPR rotates 52.32% the amount of the DP (*p* = 0.0044) and has 4.44% of the creep (*p* < 0.0001). The PBSS and IMN constructs had comparable results across all tests (*p* > 0.05). Overall, the EPR has comparable or superior mechanical stability to the DP, while the PBSS and IMN have similar mechanical properties. Together, these results can be used as a guide for surgeons to choose different implants depending on individual patient needs.

## Graphical abstract

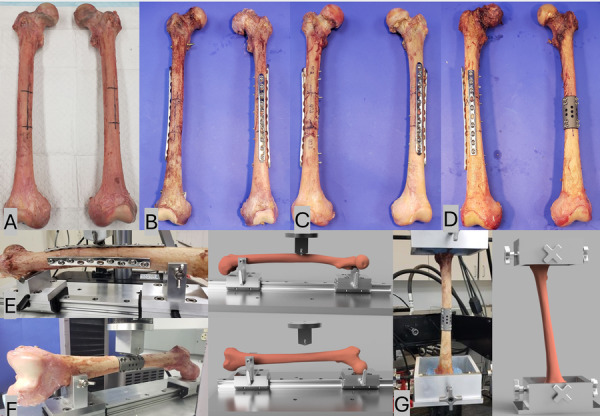

## 1 Introduction

Following joint sparing diaphyseal tumor resection, reconstructive options typically include either allograft or metallic endoprosthetic reconstruction (EPR) reconstruction. Selection of the proper intercalary reconstruction depends on individual patient risk factors, including age, prognosis, and length of diaphyseal resection. Regarding intercalary allograft reconstruction at the femur, fixation with a double plate (DP) configuration of the bone is frequently utilized, which consists of a diaphyseal allograft anchored with a medial and lateral‐based plating [1]. The location of plates is specific to the surgical approach used, the size of the defect, the reduction needed, and the surgeon's preference. This reconstruction can be combined with other biologic aids, such as vascularized fibular grafts, to promote rates of bone healing and reduce fracture risk [2]. However, these operations are used selectively, due to their technical complexity and the risk of donor site morbidity [2].

Intramedullary fixation, such as intramedullary nails (IMN) and photodynamic bone stabilization systems (PBSS), to augment allograft fixation, is reported as an alternative to DP fixation. The IMN implant acts as an internal splint for the long bone, absorbing load until bony bringing can accommodate load distribution [3]. As a result, IMN is another good candidate for limb reconstruction since it allows for potentially earlier mobilization [3, 4]. PBSS is another intramedullary stabilization approach that may minimize risks associated with traditional IMN, such as iatrogenic fracture when passing a rigid nail. Increased freedom in placement of the device start point also aids in avoiding tumor disturbance [5, 6, 7, 8]. This device utilizes an inflatable balloon that can be filled with liquid polymer. This polymer conforms to the internal geometry of the diaphysis before being cured by the application of ultraviolet light. PBSS may decrease the torsional stress seen with traditional titanium nails [7, 9]. Evidence in the trauma literature shows that it may be especially useful in stabilizing fractures in osteoporotic bone while also benefiting oncologic patients who commonly possess poor bone quality [5].

Another approach to reconstruction is intercalary EPR. This device utilizes two reamed stems that are placed on the proximal and distal ends of the defect and secured with a modular clamping junction. EPR constructs allow for immediate weight‐bearing, but can be prone to aseptic loosening [10, 11, 12]. Historically, EPRs have been utilized in older patients with poor potential for osseointegration, such as those with metastatic disease or radiated bone [13, 14]. Most studies have documented comparable Musculoskeletal Tumor Society scores between intercalary allografts and metal prostheses, especially in the long term [15, 16].

There is currently no strong evidence to support one treatment over another [11, 17, 18]. Thus, patient‐centered decision making is undertaken to select a reconstructive option that minimizes complication rates and further intervention [17]. To better understand the mechanical properties of these reconstructive approaches and to help guide surgeons on which to use for an individual patient, the present study examined the effects of axial loading, bending, and torsional forces on cadaver bones, with each construct discussed. Clinically, these stress–strain relationships in the setting of a reconstruction or fracture fixation are defined as mechanical stability, which influences surrounding tissue formation, bone remodeling, and fixation anchoring. In comparing the mechanical stability of double plating, IMN, PBSS, and EPR, it is hypothesized that the PBSS implant will be more mechanically stable than the IMN construct due to its ability to contour to the specific bone geometry in a structurally stable 7 cm defect and provide torsional stability. Additionally, it is hypothesized that the EPR constructs will have superior mechanical stability than the DP in a structurally stable 7 cm defect, due to the additional stability provided by the cemented stem insert.

## 2 Materials and Methods

### 2.1 Cadaver Dissection and Procedure

**Figure 1:**
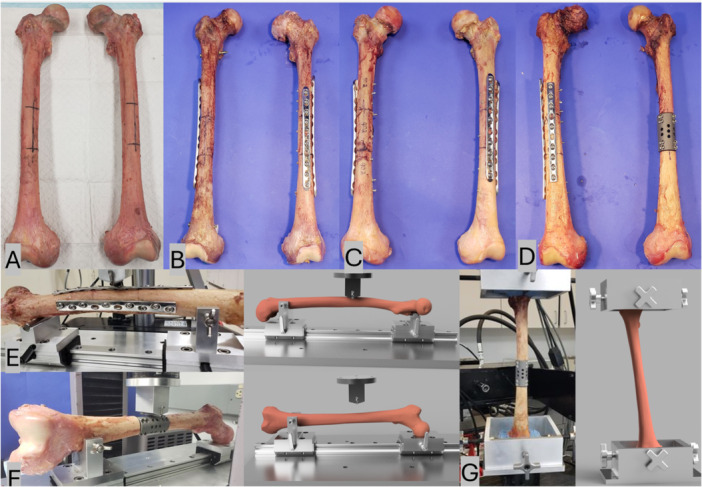
Pair matched femora prior to reconstruction (A), paired matched IMN (left) and DP (right) samples (B), paired matched PBSS (left) and DP (right) samples (C), paired matched IP (right) and DP (left) samples (D), test set up for AP bending and 3D rendering (E), test setup ML bending and 3D rendering (F), test setup axial compression and torsional testing with 3D rendering (G).

Fifteen sets of paired cadaveric femora were purchased from Science Care (Folcroft, PA), and the surrounding soft tissue was excised prior to surgical procedures. The average age of the donors was 79.4 ± 9.4 years with an average BMI of 25.6 ± 7.8. No statistical differences were found between treatment groups for age and BMI. Five paired femora (same donor) were randomly assigned to one of three comparison groups: IMN, PBSS, and EPR. Each comparison group had one femur from each pair match assigned to an intramedullary fixation construct, while the other femur from the pair was assigned to a DP construct to serve as a control and compensate for differences in size and shape of samples between donors. For the constructs that included an allograft, the segment that was removed was reused for the reconstruction. For the DP construct, femora were fixated with an 11‐hole broad plate laterally and a 10‐hole narrow plate anteriorly (DP). Each pair of matched femora was tested in axial loading, bending, and torsion. Any pairs of femora with outlier biomechanical properties (values greater than 1.5 standard deviations) were considered outliers and removed. Excluded femoral samples included one PBSS‐treated femur for cyclic bending, one IMN‐treated femur for cyclic torsion, and one EPR‐treated femur for torsion to failure. All femora were assessed under fluoroscopy during and after each procedure to ensure no skeletal defects were present and implants were inserted correctly (Figure 1).

### 2.2 Creation of Femoral Defect

All femora were measured from the head of the femur to the medial condyle, and the mid‐point was marked. From there, 3.5 cm was measured proximally and distally, and a center line was made to note the rotational relationship of all parts. Next, a 7 cm intercalary segment was cut out of the diaphysis to emulate resection. This size defect was chosen to match the largest single‐segment EPR clamping junction.

### 2.3 Plating Procedures

For the double plating, IMN, and PBSS procedures, an 11‐hole 4.5 mm broad plate was clamped to the lateral aspect of the distal segment and fixed concentrically with 3.5 mm screws. The intercalary segment was then joined and compressed to the distal segment through the plate via eccentric drilling at two points, bicortically for the double plating procedure and unicortically for the IMN and PBSS procedures. This process was repeated for the proximal segment, producing fixation at three points above and below the defect. For the DP construct, a 10‐hole 4.5 mm narrow plate was placed anteriorly and orthogonally to the first plate and fixed with 3.5 mm screws (Figure 1A–D). For the IMN construct and PBSS, a 15 mm opening was made to place a guidewire (verified on fluoroscopy) and reamed to 1–1.5 mm after cortical contact was noted. For the IMN construct, the nail was inserted, and the nail interlocks were then placed, one screw distally and proximally (Figure 1B). For the PBSS construct (implant donated from IlluminOss, East Providence, RI), the balloon was then attached and inflated under fluoroscopy, and then, using UV light, it was cured (Figure 1C).

For the EPR group [implant donated from Merete, Oakbrook Terrace, IL], the distal and proximal segments of bone were sequentially reamed from 8.5 by 1 mm until cortical contact was noted audibly. Once cortical contact was established, reaming was conducted with 0.5 mm intervals until 1.5–2 mm over the desired stem diameter. In most cases, the stem was 14 mm in diameter proximally and 16 mm distally. From here, a 130 mm stem trial was placed to determine fit. Then, two bags of PMMA cement were mixed and packed by hand into the intramedullary canal, and the final stem was pressed in. The cement was given at least 20 min to harden before the 7 cm intercalary spacer was placed and fixed with screws. Screws were tightened according to the manufacturer's instructions, and a torque limiter was utilized to prevent over‐tightening. Screws were then retightened sequentially three times as per the manufacturer's surgical technique guide. All stems used were 130 mm in overall length, with 18 mm used for clamping, and all spacer segments were 7 cm for an overall construct length of approximately 294 mm (Figure 1D).

### 2.4 Mechanical Testing

Fixated femora (Figure 1A–D) were positioned in custom‐machined fixtures with constructs attached to non‐ destructively test in cyclic anterior–posterior (AP) bending (Figure 1E) followed by medial‐lateral (ML) (Figure 1F) bending for 100 cycles at 300–800 N in load control at 0.2 Hz. For AP bending, samples were placed in a custom adjustable testing fixture. Here, the distal diaphyseal‐metaphyseal junction of the bone was secured rigidly with two set screws. The proximal end rested on a smooth roller just distal to the intertrochanteric region. For ML bending, the Distal diaphyseal‐metaphyseal junction was secured rigidly with two set screws, and the proximal femoral head was rested into a smooth concave plate to allow for motion. For both tests, gauge length was measured between the proximal contact point and the distal contact point of the bone and set screws. The change in displacement per cycle and over 100 cycles was measured. Samples were then removed from the fixtures, hydrated with saline for 10 min and subsequently potted in square molds with dental cement. Here, the zone of cement potting was marked out with the sample seated in the potting mold. Next, wood screws were placed through the epiphysis in a radial fashion to increase surface area for cement fixation. No failures of the cement/sample interface were noted during testing. After the cement set for 3 h, samples were tested in axial compression. Samples were placed into testing fixtures, ensuring that the sample was upright with the diaphysis centered under the load cell. Four set screws were then tightened in parallel to secure the sample rigidly to the bottom fixture. The proximal (top) end of the sample was left free to contact the actuating arm. Samples were preloaded to 100 N and then loaded for 100 cycles in uniaxial compression at 300–800 N at 0.2 Hz (Figure 1G). The change in displacement per cycle and over 100 cycles was measured. Samples were rehydrated, and the preload was removed for 10 min. Samples were then loaded in torsion between 6 and −6 Nm for 100 cycles (Figure 1G). This testing regimen was conducted with the same fixtures used for axial compression. Samples were again secured with four set screws. Then the actuator was lowered until the four set screws were at the level of the proximal cement block. The set screws were then tightened in parallel until the sample was secured. Care was taken to secure the sample while keeping the pre‐load below 0.25 Nm. Finally, samples were internally rotated in stable angle control to failure (maximum angle set at 140 degrees) using a single cycle of torsion. Throughout the torsion tests, change in angle per cycle and over 100 cycles were measured, as well as peak torque and corresponding angle for the torsional testing to failure. Canal geometry was measured using calipers. ML and AP bending testing were done on the electromechanical MTS Criterion (Eden Prairie, MN) using TestSuite TQ Software (Seefeld, Germany). Axial and torsion tests were completed on the servo‐hydraulic MTS Bionix (Maumee, OH), which was controlled via Station Manager. All tests were conducted at Exponent Inc. in Philadelphia, PA. Formulas used to compute axial stiffness, bending elastic stiffness, peak torque, torsional rigidity, max shear stress, shear modulus, work to failure, and modulus of toughness are outlined in Table S1.

### 2.5 Bone Geometry Calculations

Caliper measurements, implant size, and final reamer size were used to calculate the cross‐sectional area of the bone. Each bone with its respective implant required different geometrical calculations due to its different implanting techniques and the physical properties associated with them. The DP, being an intercalary technique, did not impact the bone geometry and was calculated as a hollow ellipse. The IMN and PBSS, as intramedullary techniques that filled the intramedullary cavity, were calculated as solid ellipses. The EPR was calculated as a partially hollow and partially solid ellipse, depending on the part of the bone. The hollow portions included the proximal and distal portions of the EPR implant that were unaffected bone as well as the interior of the collar; the solid portions were those filled with cement and the portions inserted into the bone as intramedullary fixation. Formulas used to compute bone geometry based on these assumed shapes were calculated in table 1 from Engesaeter et al. [19].

### 2.6 Statistical Analysis

Each IM implant was normalized to its paired DP femur, which was set to 100%. Welch's *t*‐test was performed between DP and EPR, as well as IMN and PBSS, where statistical significance was considered at *p*‐values less than 0.05. Microsoft Excel from Microsoft Corporation (Redmond, WA) was used for data organization and mathematics. Prism from GraphPad (Boston, MA) was used for performing statistical analyses.

## 3 Results

After data collection, a power analysis was performed to determine which outcomes were sufficiently powered with a sample size of five femurs per group. Upon analysis, many measures of mechanical strength for the femurs required sample sizes of 2–5 femurs per group in AP Elastic Stiffness, ML change in displacement per cycle, change in angle per cycle in cyclic torsion, torsional rigidity, max shear stress, and shear modulus, complementing the fact that statistical significance was achieved in these measures of biomechanics. Other measures required slightly larger sample sizes of 7–15 femurs in AP change in displacement per cycle, change in displacement over 100 cycles, ML change in displacement over 100 cycles, ML elastic stiffness, and change in angle over 100 cycles in cyclic torsion. Lastly, modulus of toughness, work to failure, and all axial measurements required significantly greater sample sizes of 24–173 femurs per group. While this suggests that some measures were not adequately powered, it would not be financially feasible or efficient use of cadaveric femurs to use that many femurs to adequately power the samples, especially due to the lack of clinical significance when the changes between groups are so minor. This, combined with prior studies, resulted in a chosen sample size of 5 femurs per group.

No significant differences were present in axial loading of the different types of implants (see Figure S1 and Table S2).

**Figure 2:**
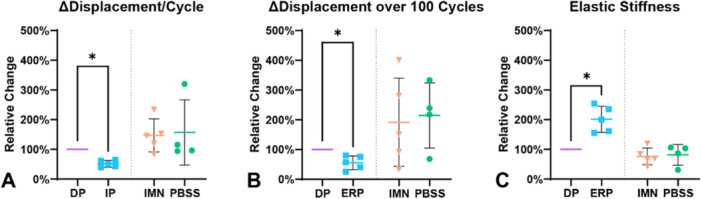
Anterior–posterior bending of the EPR was lower than the PBSS implant per cycle (A) and over 100 cycles (B); The elastic stiffness during anterior–posterior bending of the IP was higher than the double plate (C). Statistically significant relationships shown on the graph with *p*‐values less than 0.05 are denoted with an asterisk (*). Purple bars represent DP implants normalized to 100% that were pair‐matched to their respective IM implant group.

The EPR had lower relative AP displacements per cycle in bending than the DP implant (100% DP vs. 51.63% EPR, *p* = 0.0007), while there was no significant difference in displacement in IMN versus PBSS bending (147.0% IMN vs. 156.8% PBSS, *p* = 0.8788) (Figure 2A). The EPR had lower displacement over 100 cycles (100% DP vs. 55.33% EPR, *p* = 0.0126) while there was no significant difference in IMN versus PBSS bending creep (191.5% IMN vs. 214.4% PBSS, *p* = 0.7976) (Figure 2B). The EPR had a greater relative stiffness than the DP (100% DP vs. 201.3% EPR, *p* = 0.0067) while there was no significant difference in IMN versus PBSS elastic stiffness (76.1% IMN vs. 81.8% PBSS, *p* = 0.6864) (Figure 2C). Additional non‐significant data were gathered, which are found in Figure S2 and Table S3.

**Figure 3:**
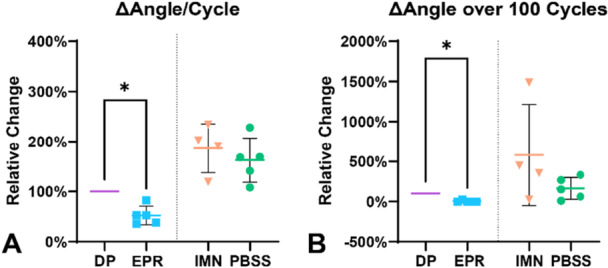
Change in angle of the EPR was less than the DP per cycle (A) and over 100 cycles (B). Statistically significant relationships shown on the graph with *p*‐values less than 0.05 are denoted with an asterisk (*). Purple bars represent DP implants normalized to 100% that were pair‐matched to their respective IM implant group.

The EPR had lower relative rotation per cycle during cyclic torsion (100% DP vs. 52.32% EPR, *p* = 0.0044) while there was no significant difference in IMN versus PBSS change in angle (186.5% IMN vs. 162.8% PBSS, *p* = 0.8277) (Figure 3A). The EPR had lower relative rotations over 100 cycles (100% DP vs. 4.44% EPR, *p* < 0.0001) while there was no significant difference in IMN versus PBSS change in torsional creep (580.5% IMN vs. 164.4% PBSS, *p* = 0.2810) (Figure 3B).

**Figure 4:**
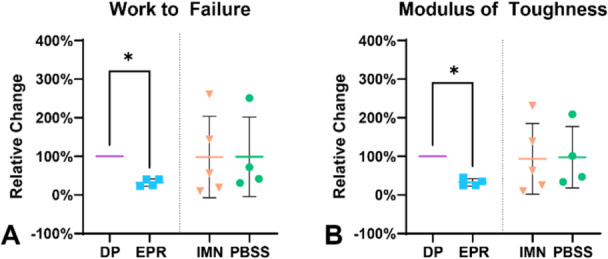
The EPR implant had lower work to failure than other implants (A). The modulus of toughness of the EPR was lower than that of the DP (B). Statistically significant relationships shown on the graph with *p*‐values less than 0.05 are denoted with an asterisk (*). Purple bars represent DP implants normalized to 100% that were pair‐matched to their respective IM implant group.

The EPR has substantially lower work to failure (100% DP vs. 32.00% EPR, *p* = 0.0007) while there was no significant difference in IMN versus PBSS change in work to failure (98.0% IMN vs. 99.0% PBSS, *p* = 0.9890) (Figure 4A). The EPR has substantially lower modulus of toughness (100% DP vs. 32.50% EPR, *p* = 0.0008) while there was no significant difference in IMN versus PBSS change in work to failure (93.6% IMN vs. 97.8% PBSS, *p* = 0.9442) (Figure 4B). Additional non‐significant data were gathered, which are found in Figure S3 and Table S4.

## 4 Discussion

This study has rigorously characterized and compared the biomechanical properties of the commonly used implants in femoral defect reconstruction. DPs have been found to have good torsional and bending rigidity, while IMN techniques have been shown to have high levels of bending without failure – both findings are consistent with this study [20, 21, 22]. Sakellariou et al. found that EPRs and IMNs performed well when loaded in axial compression. Similar to our results, this study also found good resistance to torsional stress with EPR and DP constructs, while the IMN performed worst in Torsion and three‐point bending [23].

### 4.1 Clinical Considerations for Use of Intercalary Endoprosthesis Reconstruction

The properties of the DP make it a highly rigid implant, as it consistently bent less than 1 cm and maintained high rigidity. This rigidity is shared by the EPR, except for substantially higher rigidity values and lower displacements both in cyclic torsion and AP bending. While two of the measures of torsion‐to‐failure (work to failure and modulus of toughness) show the EPR failing at lower forces, other measures are comparable. One notable example is the torsion rigidity at 448% of the DPs' torsional rigidity and does trend towards statistical significance; however, this test is slightly underpowered (*p* = 0.0652). Overall, this highlights how the EPR is more rigid than the DP. The most common failure mode in the EPR group occurred at the cement device interface, where the stem could freely rotate within the cement mantles, while the bone and stem remained intact. This is contrasted with the DP group, where fracture and screw pullout were noted.

The rigidity and mechanical stability of the EPR implant make it an attractive reconstruction option. The modularity of the device allows surgeons the option to build up a construct to make up for any resection length without the need for matching a donor segment. One drawback is the lack of osteointegration, which may support goals of long‐term durability – this highlights the importance of patient selection for reconstructive options. Alternatively, due to the rigidity and mechanical stability of a cemented EPR, this implant provides immediate stability and return to weight‐bearing without requiring biologic ingrowth. These comparisons have been reproduced in a humeral shaft model with similar results [23]. Ruggieri et al. found that while the EPR is highly modular, easy to apply, and improves preservation of the adjacent joints, complication rates as high as 33% in their series of 24 patients. Of these complications, all but one resulted from mechanical failure of the implant. Subgroup analysis indicated that resection length played a large factor in the probability of mechanical failure, with 72% (5/7) of these patients having a resection length greater than 10 cm [24]. A study by Ma et al. performed a finite‐element analysis on a free vascular graft and locking plate reconstruction for a segmental femur defect. The authors found a sharp increase in von Mises stresses at both the graft and plate when the defect length was extended to 12 cm [25]. Together, these studies highlight how increased resection length drastically increases the stress and strain, regardless of the reconstruction option.

### 4.2 Clinical Considerations for Allograft Reconstruction With Traditional Fixation Methods

The integrity and stability of the graft must be protected until the graft can achieve union. During this waiting period, fixation is achieved with traditional plating techniques, and the patient is usually non‐weight‐bearing. Dual plating has been most commonly used historically, with aseptic non‐union rates ranging from 6%–43% [26]. This construct was the second most rigid out of all the reconstructive options tested. However, there are concerns about its ability to provide axial stability, which could result in a loss of graft stability. This technique also does not allow for early return to weight‐bearing and is associated with up to 22 weeks of limited weight‐bearing, which can negatively impact patient quality of life and increase risk for morbidity such as venous thromboembolic events [26].

The IMN and PBSS implants both showed increased strain under torsion compared to either the DP or EPR, without compromising mechanical stability, as seen by the comparable work‐to‐failure and peak torque, highlighting their increased compliance. Regarding IMN fixation, it is important to consider its elastic properties. This reconstruction also has the benefit of having load‐sharing properties. These properties allow for osteointegration and potential earlier return to weight‐bearing, which is a substantial benefit to patients. Axial loading at the graft interface may also be advantageous for union.

### 4.3 Novel Allograft Fixation With PBSS Augmentation

Much like the IMN, the PBSS group was shown to be less rigid but have comparable durability compared to the EPR and DP groups. The PBSS seems to be a viable solution with comparable mechanical properties to standard IMN. This load‐sharing construct has the added benefit of contouring to the overall geometry of the final construct. An additional benefit of this method is the ability to place screws through the device after it cures and hardens. This allows for bi‐cortical fixation with added support from the polymer, which may be particularly beneficial in patients with poor bone quality. One challenge with the IMN is its fixed geometry, which leaves the overall nail diameter constrained to the narrowest portion of the remaining inner wall of the diaphysis. Moreover, in the event that a large resection length is needed, there may be a loss of isthmic fit that gives IMN its stability. The relationship of the resection with respect to the patient isthmus, need for immediate weightbearing, and local strain at the graft–host interface must be weighed against each other to determine if the IMN or PBSS implant should be used.

## 5 Limitations and Conclusions

There are limitations to this study, which stem from the fact that it was performed ex vivo. Thus, the direct clinical application of these results still needs to be validated, and long‐term outcomes of patients must be investigated to determine the efficacy of each fixation method compared to the others. The present study evaluated a 7 cm intercalary defect. Recorded defect lengths vary widely between 3 and 15 cm [24, 27, 28]. The study by Ruggieri et al. in 2011 had 11 femora with defects ranging from 10 to 14 cm; The study by Olesen et al. in 2015 had two reconstructions measuring 3.5 and 5.1 cm; The study by Olesen et al. in 2019 had five femora requiring reconstructions ranging from 7 to 11.5 cm [24, 27, 28]. This study uses a 7 cm defect standard across all femora, which may limit the generalizability of this study to other length defects. This use of a 7 cm defect was chosen as this was the longest segment available for the EPR device tested. While the system is modular and multiple segments can be added to reconstruct resected bone, we did not want this additional clamping point to increase the complexity of force distribution and failure modes. This study did not test the effects of differing shapes, sizes, and different matching between the allograft and adjacent bone, nor the effect of a clinical setting with muscle affecting these forces and stress distribution; all of which can also play a critical role in construct stability and durability. That said, the goal of this preliminary study was to reproducibly characterize the stress–strain relationship across multiple reconstructive options under multiple loading regimens.

Selecting a patient‐specific limb reconstruction technique is important for the patient and their recovery, and this study helps elucidate the baseline mechanical properties of several reconstructive options. Clinically, there is an inherent balance between providing sufficient construct rigidity to allow immediate post‐operative mobility and preserving physiologic loading to promote osseous healing. Excessively rigid constructs may prevent micro‐motion necessary to stimulate graft incorporation, while excessively compliant constructs may lead to excessive motion, implant fatigue, and loss of fixation. As such, the optimal construct depends on patient‐specific factors, including defect length, bone quality, biologic potential for healing, and desired immediate postoperative weight‐bearing. Patients with poor biologic healing potential may benefit from a more rigid construct, while those with favorable healing potential may benefit from constructs that allow controlled load transfer and encourage graft incorporation. These data may help inform future reconstruction decision‐making and promote further clinical research to determine how these differences translate into patient outcomes.

## Supporting information


**Figure S1:** Axial Loading in Femurs with implants had no significant differences between groups in displacement per cycle (Figure 1A), displacement over 100 cycles (Figure 1B), and stiffness (Figure 1C).
**Figure S2:** Medial‐Lateral Bending in Femurs with implants had no significant differences between groups in displacement per cycle (Figure 2A), displacement over 100 cycles (Figure 2B) and elastic stiffness (Figure 2C).
**Figure S3:** Femurs with implants subject to torsion until failure had no significant differences in peak torque (Figure 3A), torsional rigidity (Figure 3B), max shear stress (Figure 3C), and shear modulus (Figure 3D).
**Table S1:** Table of equations used for mechanical calculations from Engesaeter et al [21].
**Table S2:** Biomechanical measurements of the DP, IMN, PBSS, and IP implants in axial loading.
**Table S3:** Biomechanical measurements of the DP, IMN, PBSS, and IP implants in three‐point bending.
**Table S4:** Biomechanical measurements of the DP, IMN, PBSS, and IP implants in nondestructive (cyclic) and destructive (to failure) torsion.

## Data Availability

The data that support the findings of this study are available from the corresponding author upon reasonable request.
