# Supplementary material for: Intercalary Allograft Reconstruction of the Femur: A Cadaveric Comparison of Intramedullary and Plate Fixation Techniques
Source: J Orthop Res. 2026 Aug 2;44(8):e70260. doi: 10.1002/jor.70260 (PMC13428981; doi:10.1002/jor.70260)
Supplement: Supplementary file 1 — Figure S1: Axial Loading in Femurs with implants had no significant differences between groups in displacement per cycle (Figure 1A), displacement over 100 cycles (Figure 1B), and stiffness (Figure 1C). Figure S2: Medial‐Lateral Bending in Femurs with implants had no significant differences between groups in displacement per cycle (Figure 2A), displacement over 100 cycles (Figure 2B) and elastic stiffness (Figure 2C). Figure S3: Femurs with implants subject to torsion until failure had no significant differences in peak torque (Figure 3A), torsional rigidity (Figure 3B), max shear stress (Figure 3C), and shear modulus (Figure 3D). Table S1: Table of equations used for mechanical calculations from Engesaeter et al [21]. Table S2: Biomechanical measurements of the DP, IMN, PBSS, and IP implants in axial loading. Table S3: Biomechanical measurements of the DP, IMN, PBSS, and IP implants in three‐point bending. Table S4: Biomechanical measurements of the DP, IMN, PBSS, and IP implants in nondestructive (cyclic) and destructive (to failure) torsion. [file JOR-44-0-s001.docx]

| **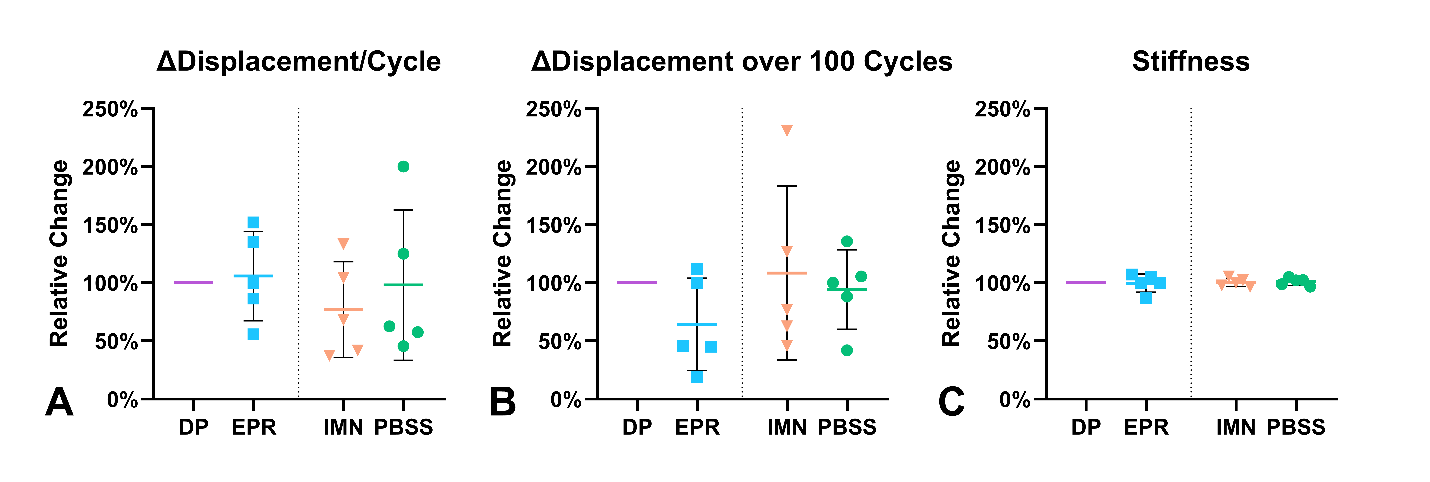**  **Figure 1. Axial Loading in Femurs with implants had no significant differences between groups in displacement per cycle (Figure 1A), displacement over 100 cycles (Figure 1B), and stiffness (Figure 1C).** No statistical significance (P-values > 0.05). Purple bars represent DP implants normalized to 100% that were pair matched to their respective IM implant group. |
| --- |

| 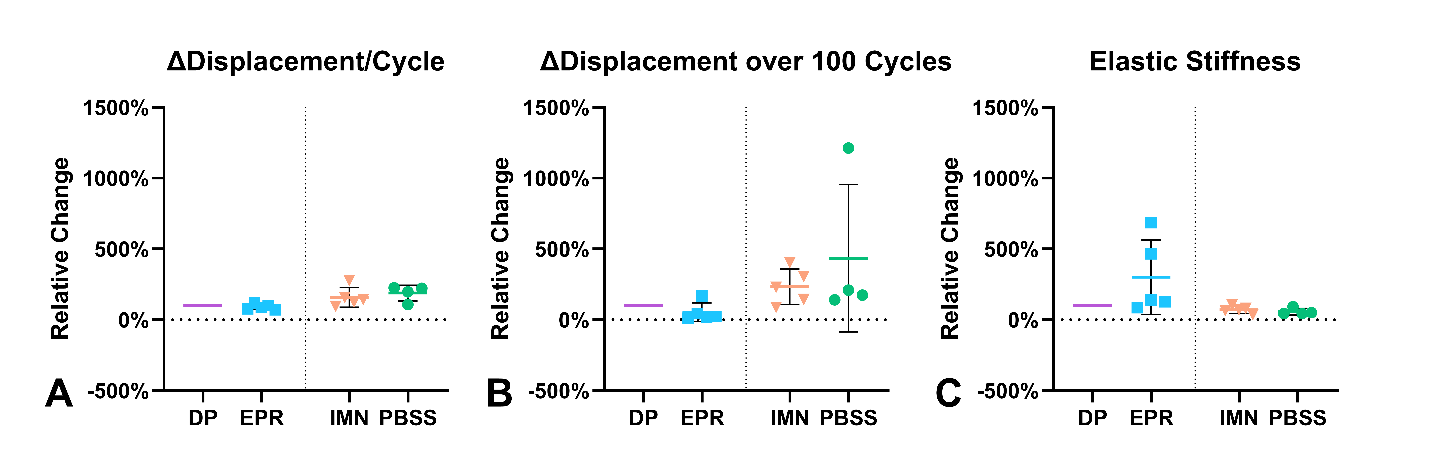  **Figure 2. Medial-Lateral Bending in Femurs with implants had no significant differences between groups in displacement per cycle (Figure 2A), displacement over 100 cycles (Figure 2B) and elastic stiffness (Figure 2C).** No statistical significance (P-values > 0.05). Purple bars represent DP implants normalized to 100% that were pair matched to their respective IM implant group. |
| --- |

| 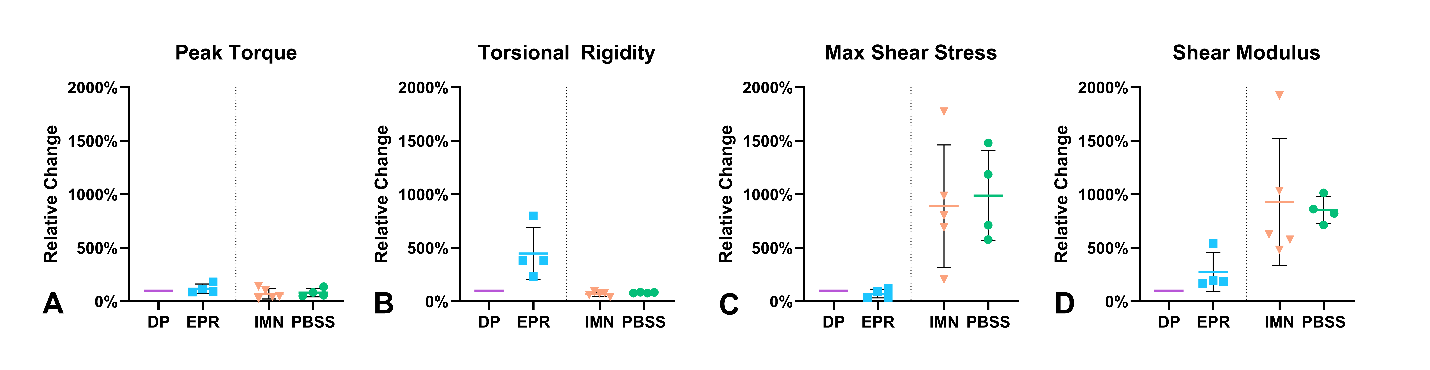  **Figure 3. Femurs with implants subject to torsion until failure had no significant differences in peak torque (Figure 3A), torsional rigidity (Figure 3B), max shear stress (Figure 3C), and shear modulus (Figure 3D).** No statistical significance (P-values > 0.05). Purple bars represent DP implants normalized to 100% that were pair matched to their respective IM implant group. |
| --- |

**Supplemental Material**

**Table 1. Table of equations used for mechanical calculations from Engesaeter et al [21].**

| **Symbols** | **Equations** |
| --- | --- |
| *Bone Geometry Equations* | |
| Maximum Interior Radius = A_i_ | Semi-major axis (a) = A_i_ + (A_o_ - A_i_)/2 |
| Maximum Outside Radius = A_o_ | Semi-minor axis (b) = B_i_ + (B_o_ - B_i_)/2 |
| Minimum Interior Radius = B_i_ | Thickness of the hollow ellipse (t) = [(A_o_ - A_i_) + (B_o_ - B_i_)] /2 |
| Minimum Outside Radius = B_o_ |  |
| Bone Length = L1 | DP Polar Moment of Inertia (J) =  ¼ * π * a * b^3 + b*a^3 - (a-t)*(b-t)^3 – (a-t)^3 * (b-t) |
| IP Solid Length = L2 | IMN and PBSS Polar Moment of Inertia (J) =  ¼ * π * [A_o_/2 * (B_o_/2)^3] |
| IP Hollow Length = L3 | IP Polar Moment of Inertia (J) =  ¼ * π * a * b * (a^2 + b^2 + L1) + (L2 * J_s_) +(L3 * J_h_) |
| Polar Moment of Inertia of the solid portion of the IP = J_s_ |  |
| Polar Moment of Inertia of the hollow portion of the IP = J_h_ |  |
| *Mechanics Equations* | |
| Force = F | Axial Stiffness = F / (L_G_ - Δx) |
| Gage Length = L_G_ | Bending Elastic Stiffness = F / Δx |
| Change in Displacement = Δx | Torsional Rigidity = L_G_ / θ_max_ |
| Max rotational angle = θ_max_ | Max Shear Stress = T * A_o_ / J |
| Polar Moment of Inertia = J | Shear Modulus = L_G_ * T / (B_i_ * J) |
| Peak Torque = T | Work to Failure (W) = θ_max_ * T |
| Cross Sectional Area = A_CS_ | Modulus of Toughness = W / (A_CS_ * L_G_) |

**Table 2. Biomechanical measurements of the DP, IMN, PBSS, and IP implants in axial loading.**

|  | Implant | DP (SD) N = 15 | IP (SD)  N = 5 | IMN (SD)  N = 5 | PBSS (SD)  N = 5 |
| --- | --- | --- | --- | --- | --- |
| Axial Loading | Δ Displacement Per Cycle (mm) | 0.3867 (0.1724) | 0.3440 (0.07092) | 0.2880 (0.1370) | 0.3100 (0.1294) |
|  | Δ Displacement over 100 Cycles (mm) | 0.2707 (0.1508) | 0.2020 (0.09257) | 0.2340 (0.1108) | 0.1580 (0.03033) |
|  | Stiffness (N/mm) | 1.193 (0.1270) | 1.267 (0.06685) | 1.156 (0.1299) | 1.164 (0.1100) |

**Table 3. Biomechanical measurements of the DP, IMN, PBSS, and IP implants in three-point bending.**

|  | Implant | DP (SD) N = 15 | IP (SD)  N = 5 | IMN (SD)  N = 5 | PBSS (SD)  N = 5 |
| --- | --- | --- | --- | --- | --- |
| Anterior-Posterior Bending | Δ Displacement Per Cycle (mm) | 0.7271 (0.2332) | 0.4160 (0.08562) | 147.0 (55.64) | 146.7 (97.48) |
|  | Δ Displacement over 100 Cycles (mm) | 0.4100 (0.2396) | 0.2500 (0.2017) | 191.5 (148.4) | 203.1 (98.29) |
|  | Elastic Stiffness (N/mm) | 743.0 (198.4) | 1239 (227.6) | 76.08 (28.43) | 84.22 (30.63) |
| Medial-Lateral Bending | Δ Displacement Per Cycle (mm) | 0.9947 (0.3878) | 0.7680 (0.2319) | 158.3 (69.27) | 233.8 (112.2) |
|  | Δ Displacement over 100 Cycles (mm) | 0.5947 (0.3145) | 0.2850 (0.4023) | 233.5 (125.2) | 907.4 (1150) |
|  | Elastic Stiffness (N/mm) | 583.6 (244.1) | 695.9 (190.6) | 71.49 (25.35) | 51.32 (25.18) |

**Table 4. Biomechanical measurements of the DP, IMN, PBSS, and IP implants in nondestructive (cyclic) and destructive (to failure) torsion.**

|  | Implant | DP (SD) N = 15 | IP (SD)  N = 5 | IMN (SD)  N = 5 | PBSS (SD)  N = 5 |
| --- | --- | --- | --- | --- | --- |
| Cyclic Torsion | Δ Angle Per Cycle (Degrees) | 2.476 (1.091) | 1.280 (0.4171) | 202.8 (55.65) | 162.8 (44.14) |
|  | Δ Angle Over 100 Cycles (Degrees) | 0.09733 (0.08031) | 0.004000 (0.008944) | 1150 (1387) | 164.4 (135.5) |
| Torsion to Failure | Peak Torque (N*m) | 55.07 (28.73) | 51.21 (11.51) | 70.60 (48.58) | 108.6 (67.73) |
|  | Torsional Rigidity (N*m/rad) | 70.60 (40.87) | 191.0 (35.88) | 68.40 (19.35) | 72.40 (19.65) |
|  | Max Shear Stress (MPa) | 14.67 (11.15) | 15.80 (3.962) | 891.0 (571.4) | 1276 (737.2) |
|  | Shear Modulus (GPa) | 3.133 (1.506) | 4.800 (1.095) | 927.4 (595.1) | 766.4 (222.0) |
|  | Work to Failure (J) | 25.24 (21.84) | 6.220 (4.409) | 98.00 (105.4) | 302.2 (463.0) |
|  | Modulus of Toughness (N/m^3) | 291403 (293801) | 298857 (162466) | 61747 (72903) | 129795 (43376) |
